# Impact of Diet-Induced Obesity and Testosterone Deficiency on the Cardiovascular System: A Novel Rodent Model Representative of Males with Testosterone-Deficient Metabolic Syndrome (TDMetS)

**DOI:** 10.1371/journal.pone.0138019

**Published:** 2015-09-14

**Authors:** Daniel G. Donner, Grace E. Elliott, Belinda R. Beck, Andrew C. Bulmer, Eugene F. Du Toit

**Affiliations:** 1 Heart Foundation Research Centre, Menzies Health Institute Queensland, Griffith University, Gold Coast, Queensland, Australia; 2 School of Allied Health Sciences, Griffith University, Gold Coast, Queensland, Australia; Stellenbosch University, SOUTH AFRICA

## Abstract

**Introduction:**

Current models of obesity utilise normogonadic animals and neglect the strong relationships between obesity-associated metabolic syndrome (MetS) and male testosterone deficiency (TD). The joint presentation of these conditions has complex implications for the cardiovascular system that are not well understood. We have characterised and investigated three models in male rats: one of diet-induced obesity with the MetS; a second using orchiectomised rats mimicking TD; and a third combining MetS with TD which we propose is representative of males with testosterone deficiency and the metabolic syndrome (TDMetS).

**Methods:**

Male Wistar rats (n = 24) were randomly assigned to two groups and provided *ad libitum* access to normal rat chow (CTRL) or a high fat/high sugar/low protein “obesogenic” diet (OGD) for 28 weeks (n = 12/group). These groups were further sub-divided into sham-operated or orchiectomised (ORX) animals to mimic hypogonadism, with and without diet-induced obesity (n = 6/group). Serum lipids, glucose, insulin and sex hormone concentrations were determined. Body composition, cardiovascular structure and function; and myocardial tolerance to ischemia-reperfusion were assessed.

**Results:**

OGD-fed animals had 72% greater fat mass; 2.4-fold greater serum cholesterol; 2.3-fold greater serum triglycerides and 3-fold greater fasting glucose (indicative of diabetes mellitus) compared to CTRLs (all p<0.05). The ORX animals had reduced serum testosterone and left ventricle mass (p<0.05). In addition to the combined differences observed in each of the isolated models, the OGD, ORX and OGD+ORX models each had greater CK-MB levels following *in vivo* cardiac ischemia-reperfusion insult compared to CTRLs (p<0.05).

**Conclusion:**

Our findings provide evidence to support that the MetS and TD independently impair myocardial tolerance to ischemia-reperfusion. The combined OGD+ORX phenotype described in this study is a novel animal model with associated cardiovascular risk factors and complex myocardial pathology which may be representative of male patients presenting with TDMetS.

## Introduction

For decades, cardiovascular disease has remained the leading cause of mortality worldwide [1]. Outside of sub-Saharan Africa (where HIV/AIDS and malaria are identified healthcare priorities) ischemic heart disease is the world’s leading cause of years-of-life-lost [2].

For several decades cardiovascular research has been performed using healthy and young, non-diseased animal models. Recent failures of cardioprotective therapies in obese insulin-resistant [3, 4], diabetic [5], metabolic syndrome-affected [6] and aged [7] animals that were otherwise successful in healthy animal models has highlighted the need for the development of animal models of disease that are representative of human clinical conditions [8–10].

The majority of laboratory-based studies investigating cardiovascular disease and myocardial tolerance to ischemia-reperfusion (I-R) are currently conducted using normogonadic models with either genetically-induced [11] or diet-induced [3, 12–15] obesity and metabolic syndrome (MetS). In the clinical setting, elderly male patients often present with both testosterone deficiency (TD) and the MetS [16, 17]. A strong and compounding association exists between metabolic syndrome and testosterone deficiency which may have significant impact on cardiovascular disease and its outcomes which is not addressed by current models.

The MetS presents as a constellation of comorbidities associated with obesity (dyslipidaemia, insulin resistance/type II diabetes) which, when present either together [18] or alone [19], increase risk of cardiovascular disease and ischemic events. Visceral adiposity is a notable component of the metabolic syndrome and is an independent predictor of the development of pre-diabetic hyperinsulinemia and an adverse cardiovascular risk profile [20].

In men, testosterone deficiency is defined by a low circulating testosterone of <12 nmol/L (<346 ng/dL) accompanied by characteristic clinical symptoms including erectile dysfunction, loss of libido and/or sarcopenia [21–23]. TD may also contribute to the increased prevalence of cardiovascular risk factors [24] and abnormal glucose metabolism [25] seen in older men, which are predictors of adverse cardiovascular outcomes [26].

The “cause and effect” relationship between low testosterone and body weight remains unresolved. However, a reciprocal interplay between the two factors has been well described [27]. Incremental increases in body mass index [28, 29] and the additional presence of the metabolic syndrome [16, 17] are associated with increased risk of developing hypogonadism in men. Additionally, circulating testosterone plays a critical role in the regulation of body composition through maintenance of muscle (lean) mass and by reducing fat mass [30] mediated, in part, by influencing stem cell differentiation [31]. Clinical evidence has repeatedly demonstrated associations between hypogonadism and measures of visceral obesity; with reduced testosterone concentrations associated with increased body mass index (BMI) [32, 33] and waist circumference [34].

Although laboratory investigations generally rely on animal models of isolated metabolic syndrome or hypogonadism, their mutual presentation in the clinical setting warrants the development of appropriate animal models of the MetS with hypogonadism, especially in the context of cardiovascular disease research.

The primary objective of the current study was to describe and compare: 1) a model of diet-induced MetS; 2) a model of orchiectomy-induced TD and 3) a combined model of diet-induced MetS with TD in male Wistar rats (TDMetS). Our results provide evidence to support the use of this robust, low-cost animal model(s) for the pre-clinical evaluation of therapies for cardiovascular disease.

## Materials and Methods

### 2.1 Animal care and model establishment

Twenty four male Wistar rats (500 ± 15 g; 12 weeks of age) were acquired from the Australian Research Centre (ARC, Adelaide, South Australia). Rats were housed under an artificial 12-hour day/night lighting cycle at a constant temperature of 21°C (40% humidity) and provided with *ad libitum* access to fresh food and water. At twelve weeks of age, rats were randomly assigned into two groups and fed either a standard rat chow (CTRL) or obesogenic diet (OGD) for the remainder of the study. At twenty weeks of age, rats from each group were randomly assigned into two sub-groups to undergo either a sham operation or orchiectomy (ORX; n = 6/group). At forty weeks of age, animals were euthanized by rapid heart excision at the completion of myocardial tolerance to ischemia-reperfusion experiments. All animals (n = 6/group) were used in all experimental assessments except for echocardiography assessments of cardiac structure and function where a randomly assigned sub-group of animals from each group (n = 4/group) were assessed. All animal procedures and assessments of tissue and sera were performed in the laboratory and the order in which animals from different groups were assessed was randomly assigned for each procedure.

Following sham and ORX operations, buprenorphine (10 μg/kg per day, i.m.) and enrofloxacin (5 mg/day, i.p.) were administered for the management of post-operative pain and infection, respectively; and all efforts were made to minimize animal suffering.

#### 2.1.1 Ethics statement

All animal experimentation was approved and performed in accordance with the guidelines of the Animal Ethics Committee of Griffith University (Permit Number: MSC/01/11) and the *Australian code of practice for the care and use of animals for scientific purposes*.

### 2.2 Diet composition and macronutrient consumption

Rats were provided *ad libitum* access to either a control diet (Meat Free Rat and Mouse Cubes, Specialty Feeds, Western Australia; CTRL) or a high fat obesogenic diet for a period of 28 weeks. Previously in our laboratory, this obesogenic diet has been shown to induce hyperphagia, increased visceral fat accrual and insulin resistance [35, 36].

Daily consumption of both standard rat chow and obesogenic diet was measured over a period of three days, six weeks after orchiectomies were performed. Macronutrient composition of each diet was determined (Queensland Food Laboratories, Springwood, QLD, Australia). Daily energy and macronutrient (fat [total, saturated, monounsaturated, polyunsaturated], protein and carbohydrate [total and sugars]) consumption was approximated for each group, considering the mass of diet consumed and the analysis of diet composition.

### 2.3 Orchiectomy procedures for induction of hypogonadism

Animals were anesthetised (2.5% Isoflurane in 1 L/min 100% medical grade O_2_) in the morning at 9 am ± 1 hr. Maintenance of anaesthesia was monitored by assessing the pedal withdrawal reflex at 5 min intervals. Testes were removed via cautery and the peri-scrotal cavity was closed using 6–0 prolene sutures. The external scrotal incision was subsequently closed using 4–6 stainless steel wound clips. Animals designated as normogonadic controls (CTRL) and normogonadic obese (OGD) underwent a sham operation where an external scrotal and peri-scrotal incision was made before closing both wounds as described above.

### 2.4 Body mass and composition assessments

Total body mass was measured at zero, four, eight, twelve, sixteen, twenty and twenty-six weeks after the commencement of the feeding program.

At 38 weeks of age (twenty-six weeks after the commencement of feeding), Dual-energy X-ray Absorptiometry (DXA; XR-36 Quickscan densitometer, software version 2.5.3a, Norland Medical Systems, Inc., USA, Host/Scanner: 4.2.4/2.3.1) scans were performed for each animal. Each scan was performed at a high resolution (1.5 x 1.5mm, speed of 6mm/s) in “small animal mode” in order to accurately measure fat mass and lean mass. In order to perform the scans, rats were sedated in the morning at 9 am ± 1 hr with a dual preparation i.p. injection of 50 mg/kg ketamine (Ketamil, Troy Laboratories, Australia) and 3 mg/kg xylazine (Ilium xylazil-20, Troy Laboratories, Australia). All scanning was performed within 10 minutes of sedation and all animals had recovered from sedation within 30 minutes.

Following animal sacrifice, retroperitoneal, epididymal and visceral fat pads were excised to quantify visceral fat content and fat distribution in each animal. Subcutaneous fat mass was determined by calculating the difference between total fat mass (quantified by DXA) and visceral (excised) fat mass.

### 2.5 Assessment of cardiac structure and function using echocardiography

At 39 weeks of age, rats were anaesthetised (2.5% isoflurane in 100% medical grade O_2_, 1L/min) in the morning at 9 am ± 1 hr; and anterior and posterior left ventricular wall thicknesses were assessed throughout a typical cardiac cycle using ultrasound (Model 710b probe and Vevo 770, Visualsonics Inc., Ontario, Canada) with M-mode analysis of both short-axis and longitudinal views. Left ventricular volumes were derived using the algorithms provided by the manufacturer. Stroke volume (SV) was derived from pulsed wave Doppler assessment of the aortic volume time integral and cross sectional area product of the left ventricular outflow tract. Cardiac output (CO) was calculated using the product of heart rate and SV.

Preliminary measures of variance in cardiac structure and function indicate that n = 4 is sufficiently powered to reveal differences between groups.

### 2.6 Serum lipids, glucose and insulin assessments

At sacrifice, whole blood was collected from each animal and kept on ice at 4°C for 30 minutes before centrifugation at 1200 g for 10 minutes. Serum was transferred to Eppendorf tubes for storage at -80°C for later analysis.

Serum samples were thawed and prepared for multi-analyte analysis (COBAS Integra 400, Roche, Switzerland). All analytes of interest (total cholesterol, triglycerides and glucose) were quantified within an hour of sample thawing. Concentrations of each analyte were quantitated using calibrator for automated systems/lipids (CFAS, Roche Diagnostics, Switzerland) in addition to validation using commercially prepared quality control specimens (Precicon Control Clin Chem. Multi 1 and 2; Roche Diagnostics, Switzerland).

Serum insulin was quantified using a commercial homogenous time-resolved fluorescence (HTRF) kit (CISBIO, Codolet, France) and read by an Artemis TR-FRET microplate reader (Cosmo Bio, USA).

Impaired insulin sensitivity (i.e. insulin resistance) was routinely assessed using the homeostatic model assessment of insulin resistance (HOMA-IR) [37, 38]. HOMA-IR values were calculated using the formula: [*Glucose (mg/dl) x Serum Insulin (μU/mL)*] */ 405*.

### 2.7 Testicular mass assessments and circulating sex hormone quantitation

Immediately following sacrifice of SHAM-operated animals, each pair of testicles was surgically excised and weighed. A combined weight of each pair of testicles was reported for each animal.

Serum testosterone concentrations were quantified using homogenous time-resolved fluorescence (HTRF) kits (CISBIO, Codolet, France) and read by an Artemis TR-FRET microplate reader (Cosmo Bio, USA).

### 2.8 In vivo myocardial tolerance to ischemia-reperfusion assessments

At 40 weeks of age, rats were anesthetised (sodium pentobarbital, 60 mg/kg i.p.) in the morning at 8 am ± 1 hr, intubated and ventilated (Harvard Instruments, Model 683) before being placed on an adjustable heating pad to maintain a core temperature of 36–37°C, as monitored by rectal thermometer (Model 52II, Fluke Corporation, Everett, Western Australia). Maintenance of anaesthesia was confirmed by assessing pedal withdrawal reflexes at 10 min intervals. A thoracotomy and reversible left anterior descending (LAD) coronary artery ligation were performed as described previously [39]. The LAD coronary artery was occluded for 45 min followed by 120 min of reperfusion initiated by ligature release. At the end of reperfusion hearts were rapidly excised and whole blood was collected from the thoracic cavity. The primary outcome of the current study, levels of serum cardiac-specific Creatine Kinase (CK-MB), was quantified using spectrophotometry (COBAS Integra 400, Roche, Switzerland) and all assessments were performed in duplicate.

Power analysis of variance in previous and preliminary studies indicate that n = 6 is sufficient for detecting differences in serum cardiac-specific Creatine Kinase (CK-MB) between groups (multiple groups contrasted in a single study).

### 2.9 Statistics

Although animals were housed in pairs, each rat was measured and assessed as a single experimental unit for statistical analyses. Two-way ANOVA and Fisher’s LSD test assessed differences between groups. For regression analyses, Pearson’s correlation coefficients were computed assuming Gaussian distribution. A value for p<0.05 was considered significant.

## Results

### 3.1 Diet composition and daily macronutrient consumption

**Fig 1:**
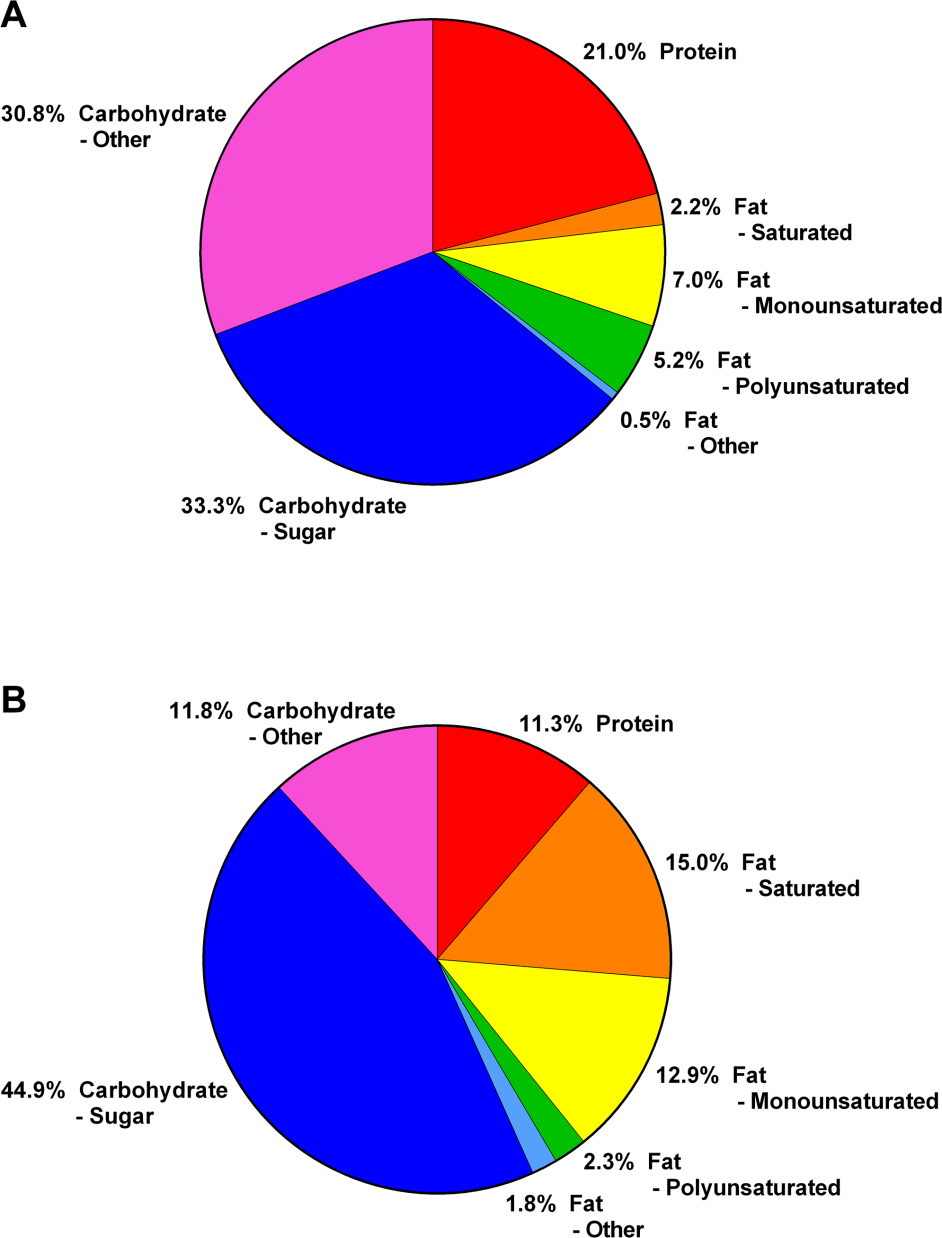
Macronutrient composition of animal diets. (A) Standard rat chow. (B) Obesogenic diet. Values expressed as a percentage of the total calorie content contributed by all macronutrients.

The obesogenic diet (OGD) used in the present study (Table 1) contained greater relative amounts of sugars, saturated and monounsaturated fat; and reduced complex carbohydrate, protein and polyunsaturated fat compared to the standard rat chow diet provided to CTRLs. Sugars contributed almost half (44.9%) and fats almost a third (32.0%) of the total calories provided by the OGD (Fig 1). In contrast, the standard chow diet provided one third of its calories from sugars (33.3%) and only 14.0% from fats.

**Table 1:** Nutritional composition of animal diets (per 100 g dry weight).

	Control Diet	Obesogenic Diet	Fold-change
	(CTRL)	(OGD)	(OGD vs. CTRL)
Energy (kJ)	1844	2047	1.11
Protein (g)	22.85	13.78	0.60
Fat-Total (g)	7.26	17.29	2.38
-Saturated (g)	1.06	8.11	7.63
-Monounsaturated (g)	3.42	6.99	2.04
-Polyunsaturated (g)	2.50	1.24	0.50
-Trans (g)	<0.1	<0.1	
Carbohydrate (g)	69.93	69.04	0.99
-Sugar (g)	36.34	54.62	1.5
Sodium (mg)	1632.96	1336.31	0.82

No significant difference in macronutrient consumption was observed when comparing orchiectomised with non-orchiectomised groups (i.e. CTRL vs. ORX; or OGD vs. OGD+ORX, p>0.05).

**Fig 2:**
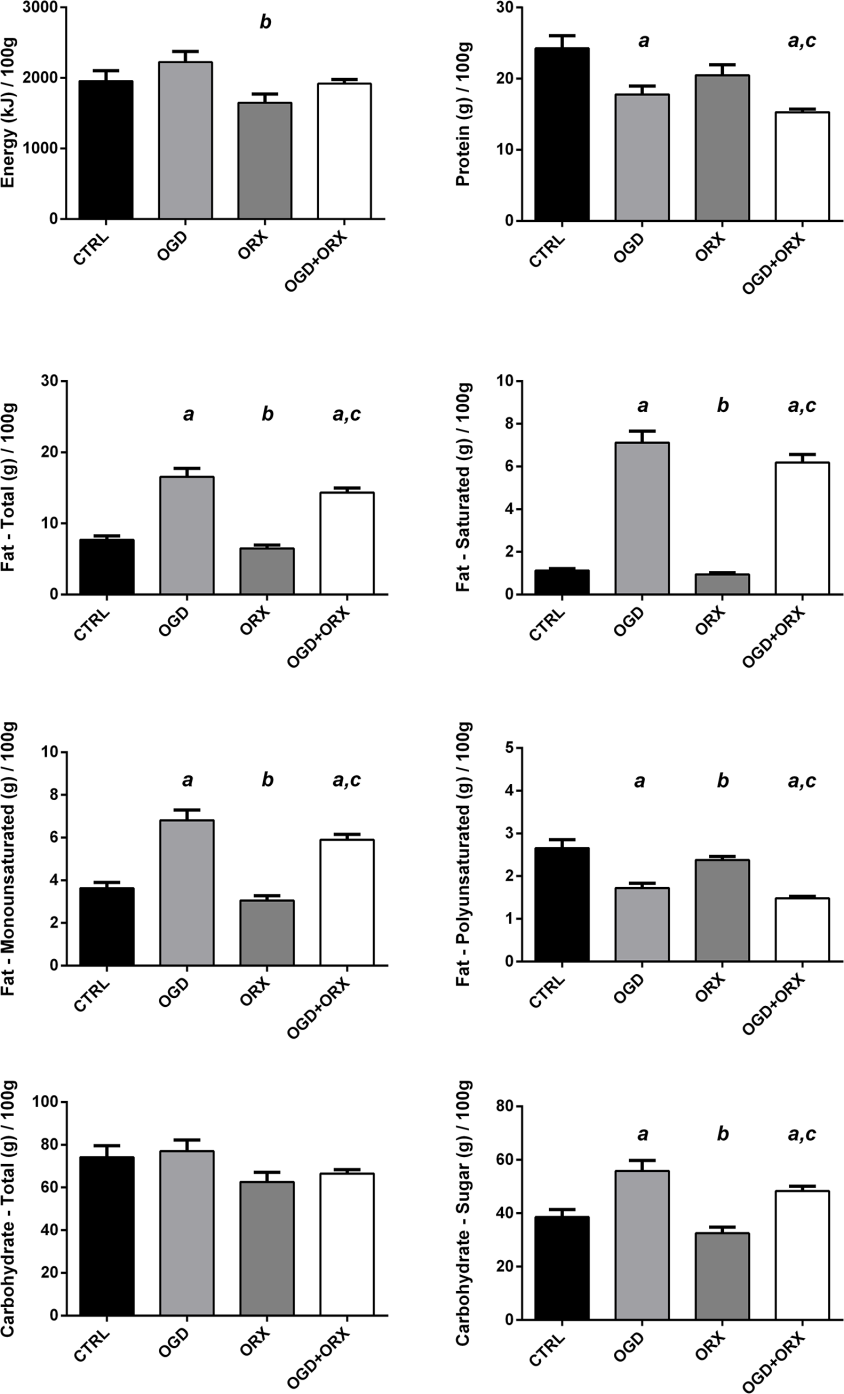
Daily energy and macronutrient intake. CTRL–control, OGD–obesogenic diet-fed, ORX–orchiectomised. Letters denote significant differences between respective groups, p<0.05 (^a^ vs. CTRL, ^b^ vs. OGD, ^c^ vs. ORX).

Both OGD and OGD+ORX animals consumed more total fat (214 and 186%), saturated fat (613 and 548%), monounsaturated fat (187 and 163%) and sugars (145 and 125%); and less protein (27 and 37%) and polyunsaturated fat (35 and 44%) than CTRL animals (p<0.05, Fig 2). Total carbohydrate consumption was similar in all four groups.

### 3.2 Body mass, body composition and fat distribution

**Fig 3:**
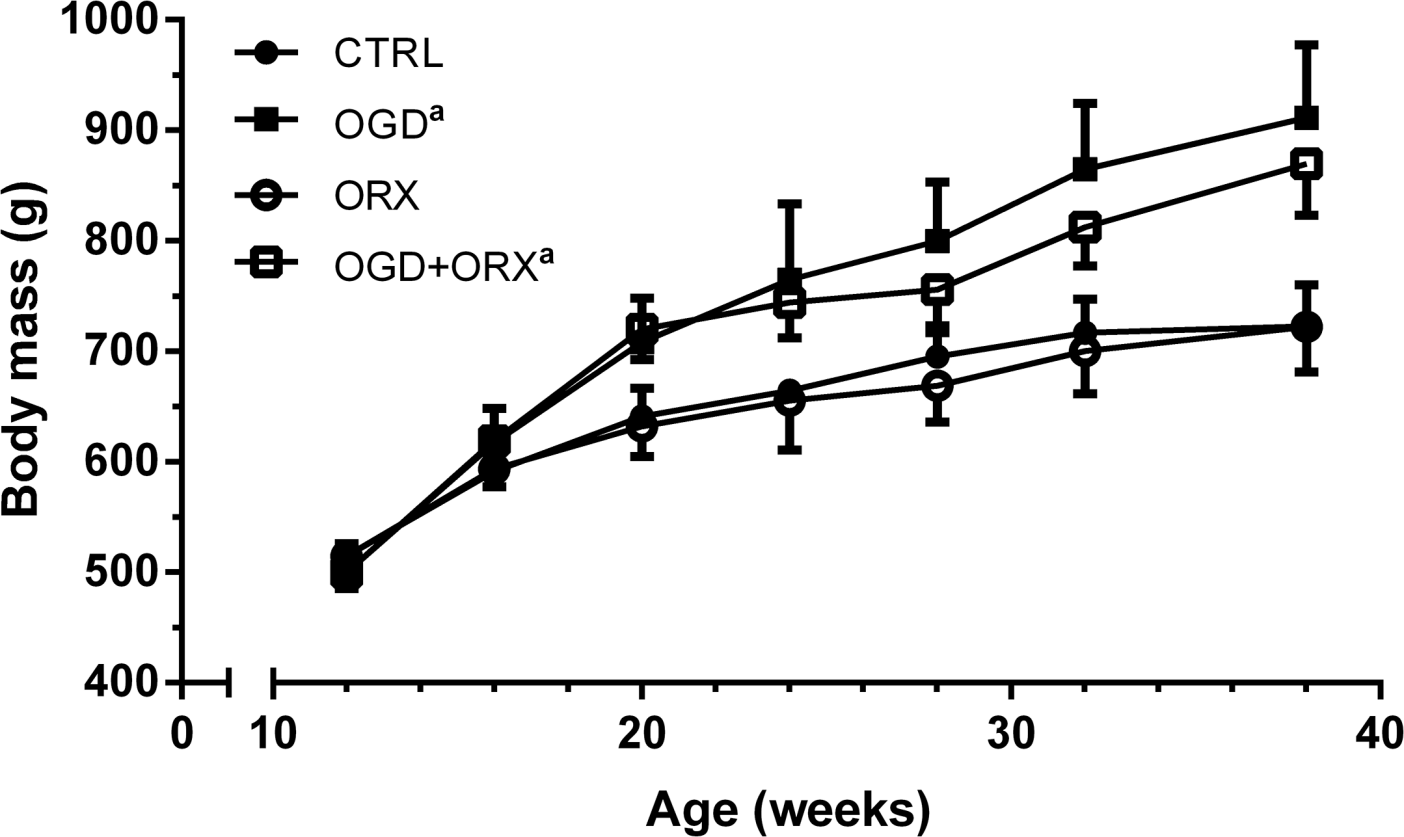
Body mass measurements. CTRL–control, OGD–obesogenic diet-fed, ORX–orchiectomised. Letters denote significant differences from respective groups at the final time point (38 weeks), p<0.05 (^a^ vs. CTRL, ^b^ vs. OGD, ^c^ vs. ORX).

All animals from each group (n = 6/group) were included in assessments of body mass, body composition and fat distribution. At 38 weeks of age, total body mass was increased in OGD and OGD+ORX animals (26 and 20% heavier, respectively; p<0.05) when compared to the CTRL group (Fig 3). In the OGD animals, body mass gain was associated with a 72% increase in total fat mass (p<0.05, Table 2) while in the OGD+ORX animals, the increase in total body mass was attributed to an 86% increase in total fat mass and a concomitant 18% decrease in lean mass (p<0.01 vs. CTRL).

**Table 2:** Biometric analysis. CTRL–control, OGD–obesogenic diet-fed, ORX–orchiectomised. Letters denote significant differences between respective groups, p<0.05.

	CTRL	OGD	ORX	OGD+ORX
	*n = 6*	*n = 6*	*n = 6*	*n = 6*
Body mass (g)	723 ± 38	911 ± 66^a^	722 ± 41^b^	870 ± 47^a^ ^,^ ^c^
Fat mass (g)	267 ± 33	491 ± 72^a^ ^,^	285 ± 49^b^	497 ± 49^a^ ^,^ ^c^
- Subcutaneous (g)	224 ± 30	401 ± 61^a^	237 ± 46^b^	423 ± 43^a^ ^,^ ^c^
- Visceral (g)	42 ± 4	90 ± 13^a^	48 ± 4^b^	74 ± 8^a^ ^,^ ^c^
Lean mass (g)	456 ± 16	420 ± 17	437 ± 33	372 ± 6^a^ ^,^ ^b^ ^,^ ^c^
Total cholesterol (mM)	0.98 ± 0.09	2.31 ± 0.24^a^	1.40 ± 0.11^b^	2.48 ± 0.25^a^ ^,^ ^c^
Triglycerides (mM)	1.08 ± 0.19	2.52 ± 0.24^a^	0.96 ± 0.19^b^	3.05 ± 0.30^a^ ^,^ ^c^
Testicular mass (mg)	3827 ± 94	3299 ± 191^a^	-	-
Epididymal mass (mg)	615 ± 13	549 ± 22^a^	-	-
Testosterone (ng/dL)	168 ± 18	136 ± 14	24 ± 5^a^ ^,^ ^b^	10 ± 1^a^ ^,^ ^b^ ^,^ ^c^

^a^ vs. CTRL

^b^ vs. OGD

^c^ vs. ORX

Overall body composition was similar between the CTRL and ORX; and the OGD and OGD+ORX groups (both p>0.05; Table 2).

**Fig 4:**
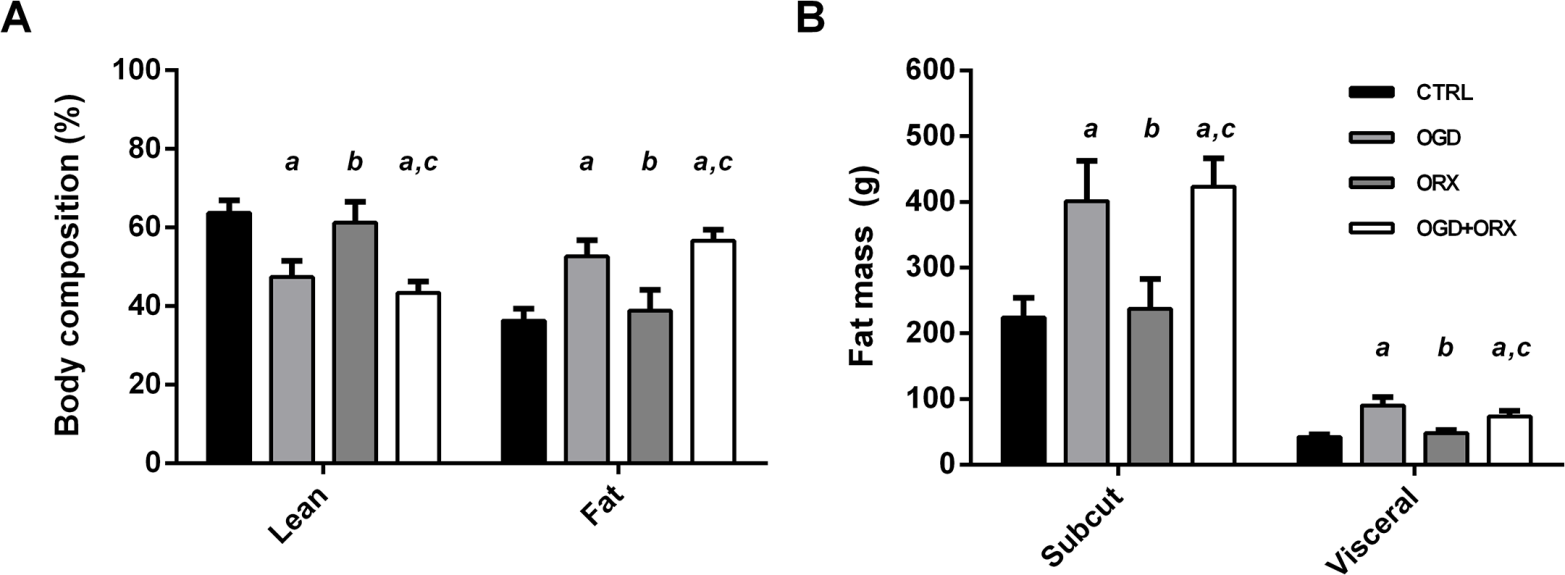
Body composition analysis. (A) Body composition expressed as a percentage of total body weight. (B) Subcutaneous and visceral fat distribution. CTRL–control, OGD–obesogenic diet-fed, ORX–orchiectomised. Letters denote significant differences between respective groups, p<0.05 (^a^ vs. CTRL, ^b^ vs. OGD, ^c^ vs. ORX).

Fat constituted the majority of total body mass in the OGD (53 ± 4%) and OGD+ORX (57 ± 3%) groups, which were significantly greater than in CTRLs (36 ± 3%, both p<0.05; Table 2 and Fig 4A). The ratio of subcutaneous:visceral fat mass was similar between control diet- and OGD-fed groups (approximately 5:1; Table 2) and neither subcutaneous nor visceral fat weights were affected by the orchiectomy in either the control or OGD-fed animals (p>0.05, Fig 4B). However, subcutaneous fat mass was approximately 80% greater; and visceral fat mass 115% greater in the OGD group compared to CTRLs (p<0.01, Table 2 and Fig 4B). Similar to the OGD group, the body composition of the combined OGD+ORX model was 57 ± 3% fat mass (43 ± 3% lean mass) with subcutaneous fat mass 87% and visceral fat mass 76% greater than in CTRLs (p<0.05, Table 2).

### 3.3 Fasting serum lipids, glucose and insulin

All animals from each group (n = 6/group) were included in assessments of fasting serum lipids, glucose and insulin. At the end of the study (40 weeks of age), serum cholesterol levels in OGD and OGD+ORX animals were 2.4-fold and 2.5-fold greater than those in CTRLs (p<0.05, Table 2). In the OGD animals, circulating triglyceride concentrations were also more than double those in CTRL and ORX animals (2.3-fold and 2.6-fold greater, respectively; p<0.01).

However, no significant differences in serum lipids were observed between CTRL and ORX animals (p>0.05).

**Fig 5:**
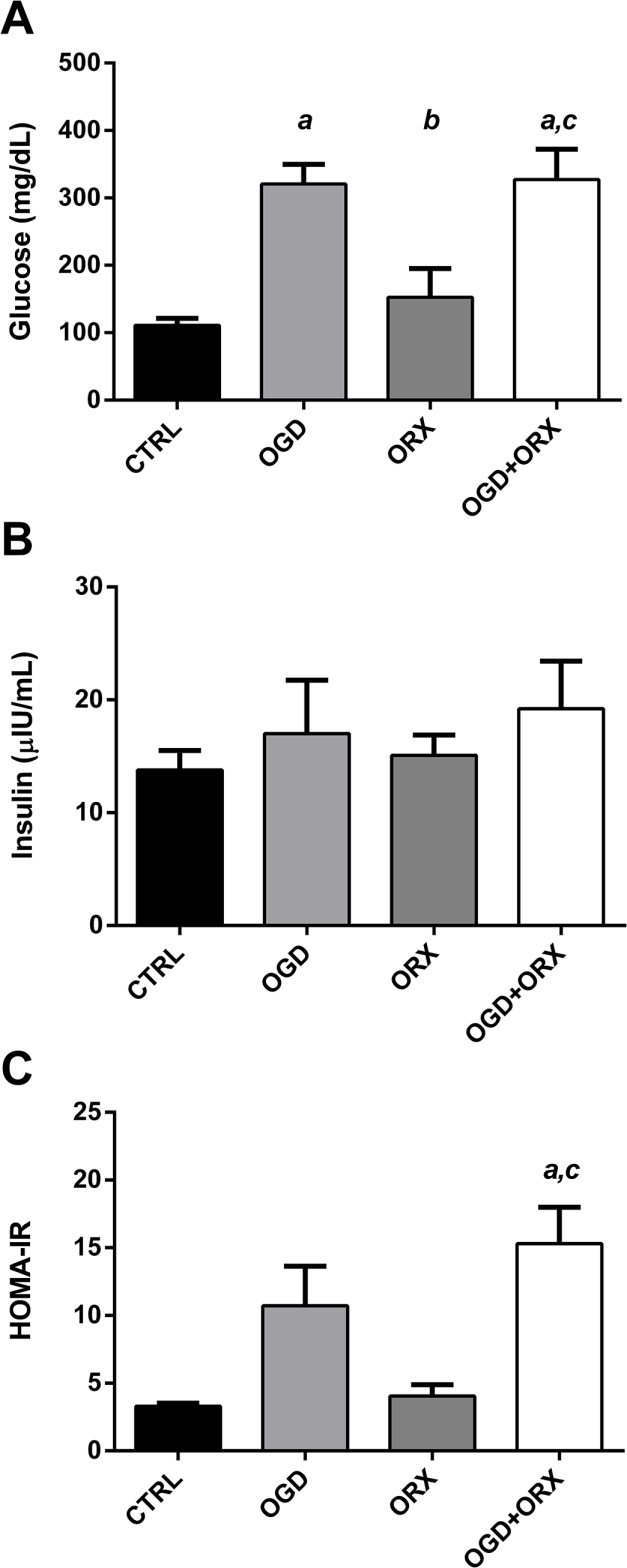
Homeostatic Model Assessment of Insulin Resistance (HOMA-IR). (A) Serum glucose concentrations. (B) Serum insulin concentrations. (C) Calculated HOMA-IR scores (in arbitrary values). CTRL–control, OGD–obesogenic diet-fed, ORX–orchiectomised. Letters denote significant differences between respective groups, p<0.05 (^a^ vs. CTRL, ^b^ vs. OGD, ^c^ vs. ORX).

Glucose concentrations were 110.9 ± 10.4 mg/dL in CTRL animals and were unaffected by ORX (p>0.05, Fig 5A). Glucose concentrations were approximately 3-fold greater in both OGD-fed groups (OGD: 320.6 ± 29.1; OGD+ORX: 327.3 ± 45.0 mg/dL, p<0.001) compared to CTRL animals. Although insulin levels were similar between all groups (Fig 5B), the resultant HOMA-IR values were 4.6-fold greater in OGD+ORX rats (15.3 ± 2.7 vs. 3.3 ± 0.2 in CTRLs, p<0.01; Fig 5C).

### 3.4 Testicular mass and circulating testosterone levels

All animals from each group (n = 6/group) were included in assessments of testicular mass and circulating testosterone. The wet weights of testes collected from OGD animals at sacrifice were 14% less than CTRLs (p<0.05, Table 2). Epididymal mass was also reduced in the OGD group by 11% (p<0.05).

Orchiectomy reduced serum testosterone concentrations in standard chow- and OGD-fed animals by approximately 86 and 93%, respectively (compared to CTRLs, p<0.001, Table 2).

### 3.5 Cardiac structure and function

For each group, four animals had been randomly assigned for assessment of cardiac structure and function and all assigned animals were included in these assessments (n = 4/group). No significant differences in left ventricular dimensions were observed between any groups during systole or diastole (p>0.05). Left ventricular (LV) mass was reduced with ORX in both ORX (15% lighter than CTRL, p<0.05) and OGD+ORX (12% lighter than OGD, p<0.05) groups (Table 3).

**Table 3:** Echocardiography analysis. CTRL–control, OGD–obesogenic diet-fed, ORX–orchiectomised. Letters denote significant differences between respective groups, p<0.05.

	CTRL	OGD	ORX	OGD+ORX
	*n = 4*	*n = 4*	*n = 4*	*n = 4*
*Dimensions (mm)*				
Anterior wall–diastole	2.12 ± 2.23	2.53 ± 0.14	1.91 ± 0.09	2.13 ± 0.08
Anterior wall–systole	3.74 ± 0.25	3.9 ± 0.05	3.26 ± 0.15	3.58 ± 0.11
Posterior wall–diastole	1.88 ± 0.13	1.94 ± 0.08	1.82 ± 0.09	1.99 ± 0.08
Posterior wall–systole	3.34 ± 0.32	3.46 ± 0.24	3.20 ± 0.22	3.27 ± 0.08
*Function*				
EF (%)	77.2 ± 6.9	78.2 ± 2.5	75.7 ± 3.2	75.1 ± 3.9
FS (%)	50.2 ± 7.8	49.2 ± 2.4	46.6 ± 3.1	46.8 ± 3.8
SV (μL)	822.1 ± 78.9	982.9 ± 56.7	854.9 ± 92.1	970.7 ± 101.8
CO (mL / min)	287.9 ± 34.1	371.2 ±21.5	302.8 ± 32.3	366.4 ± 50.0
LV mass (mg)	1347 ± 47.2	1360 ± 73.2	1134 ± 6.9^a^ ^,^ ^b^	1203 ± 13.5^a^ ^,^ ^b^

^a^ vs. CTRL

^b^ vs. OGD

Cardiac function, assessed by ejection fraction (EF %), fractional shortening (FS %), stroke volume (SV) and cardiac output (CO), was similar between all groups (all p>0.05, Table 3).

### 3.6 Serum markers of myocardial damage during ischemia-reperfusion

**Fig 6:**
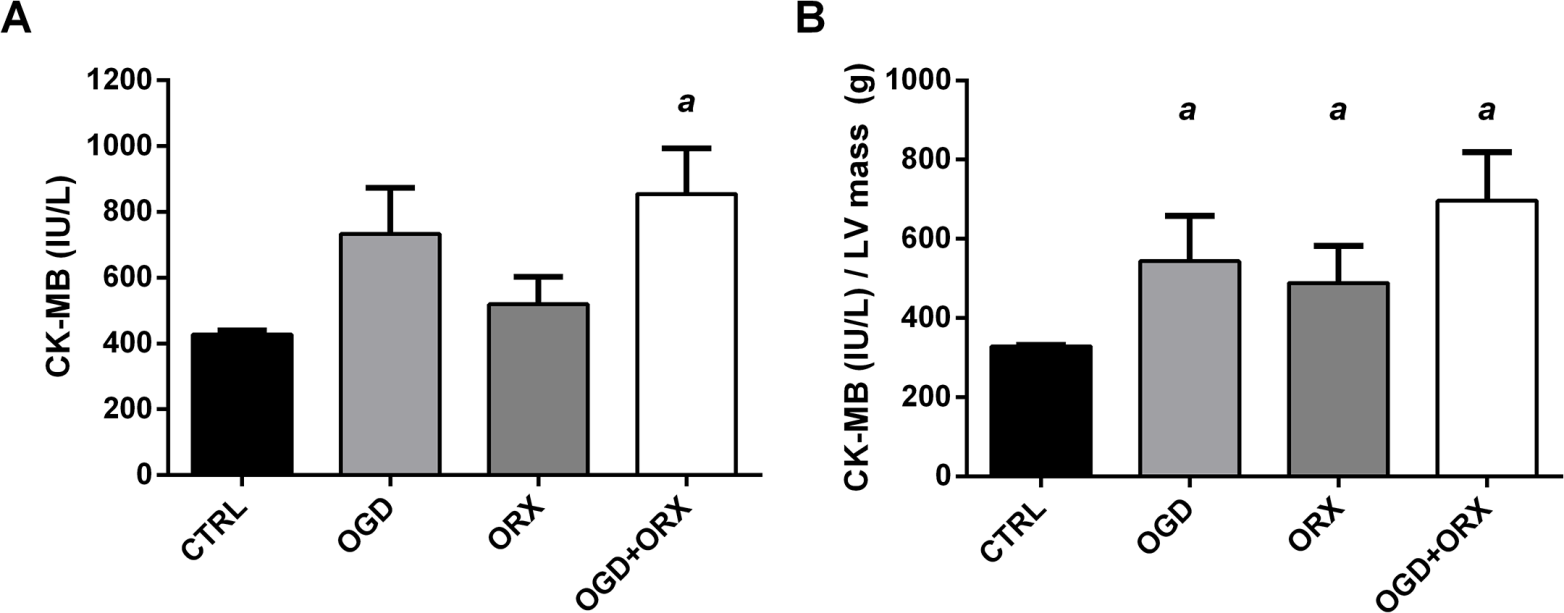
Cardiac-specific Creatine Kinase (CK-MB) levels. (A) Serum concentration. (B) Serum concentration expressed relative to left ventricular (LV) mass. CTRL–control, OGD–obesogenic diet-fed, ORX–orchiectomised. Letters denote significant differences between respective groups, p<0.05 (^a^ vs. CTRL, ^b^ vs. OGD, ^c^ vs. ORX).

Serum CK-MB levels were measured in serum obtained from all animals following the *in vivo* ischemia-reperfusion experiments (n = 6/group; Fig 6). The ORX group had 21.4% greater and the OGD group 41.3% greater CK-MB levels compared to CTRLs, however neither of these differences were significant (p>0.05) (Fig 6A). The CK-MB levels in OGD+ORX animals were 99.7% greater than CTRLs (854.2 ± 139.7 vs. 427.8 ± 13.0 IU/L in CTRL, p<0.05).

When CK-MB levels were corrected for left ventricular mass, OGD (543.7 ± 115.3 IU/L/g), ORX (488.5 ± 161.7 IU/L/g) and OGD+ORX (696.4 ± 123.0 IU/L/g) animals had greater CK-MB than CTRLs (329.0 ± 2.8 IU/L/g, both p<0.05; Fig 6B).

## Discussion

**Fig 7:**
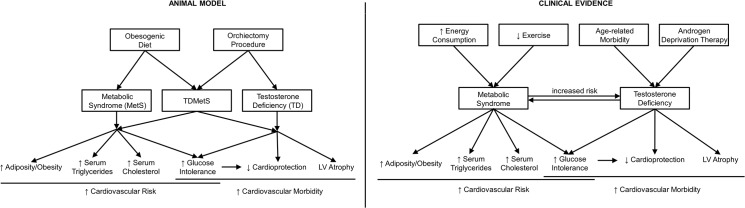
Summary of the animal model and clinical evidence of Testosterone-deficient Metabolic Syndrome (TDMetS). *The animal model* described in the present study received an obesogenic diet which induced the metabolic syndrome (MetS) by increasing adiposity, circulating triglycerides and cholesterol; and contributing to glucose intolerance. Orchiectomy procedures induced testosterone deficiency (TD) which impaired cardioprotection and also contributed to glucose intolerance in these animals. The combined model of testosterone-deficient metabolic syndrome (TDMetS) demonstrated all characteristics described in both the MetS and TD models. *Clinical evidence* supports the strong associations between MetS and TD and the impact of the combined TDMetS phenotype which is comprehensively represented in our animal model and results in an adverse cardiovascular risk profile and a complex myocardial pathology.

The current study characterised and compared three rat models of disease that mimic conditions of male patients presenting with: 1) the metabolic syndrome (MetS), or 2) testosterone deficiency (TD); or 3) testosterone-deficiency and the metabolic syndrome (TDMetS) with consequent cardiometabolic disease. Our findings with each individual model demonstrated that: 1) the obesogenic diet (OGD) increased body mass, fat mass, total circulating cholesterol and triglycerides (hallmarks of the metabolic syndrome); 2) orchiectomy (ORX) induced reductions in circulating testosterone, which was associated with atrophy of the left ventricle (LV); and 3) most importantly, the combination of both the obesogenic diet and orchiectomy resulted in a similarly overweight, overtly hypertriglyceridemic and hypercholesterolemic phenotype as in model 1 but with additional abnormalities. These animals had low testosterone, sarcopenia, hyperglycaemia, left ventricular atrophy and compromised tolerance to myocardial ischemia-reperfusion (compared to CTRL littermates; Fig 7).

A large body of evidence supports the association between the MetS, TD and cardiovascular risk factors which has prompted recommendation that subjects with components of the MetS be screened for hypogonadism in future clinical research [40]. The current study provides data demonstrating that models of testosterone deficiency combined with diet-induced metabolic syndrome are phenotypically distinct from existing models which may not represent existing cohorts of aged and obese males. This study also describes the characteristics of a novel animal model of TDMetS which should be considered in future cardiovascular disease research.

### 4.1 Rationale for a model of testosterone-deficient metabolic syndrome (MetS)

Basic laboratory-based investigations of cardiovascular disease are currently conducted in animal models of either obesity [12, 13] or hypogonadism [41, 42] independently of one another. However, the strong association between these two pathologies frequently result from a reciprocal aetiology and the clinical presentation of the two phenotypes together [28].

The MetS is characterized by visceral obesity, dyslipidaemia and insulin resistance/diabetes [43] which is caused by excessive food consumption [44], inadequate exercise [45] and testosterone deficiency in males [46, 47]. The MetS and its components have been strongly linked to elevated cardiovascular risk, morbidity and mortality [18, 48] and are universally accepted as the leading causes of cardiovascular disease worldwide.

The diagnosis of TD is defined by low circulating testosterone in males (<12 nmol/L or <346 ng/dL) and occurs frequently with increasing age [28] and with other age-related co-morbidities [32]. The metabolic syndrome [49] and its individual components [50–53] are also strongly associated with TD while androgen deprivation therapy (for prostate cancer treatment) also leads to metabolic dysregulation [54]; adverse outcomes from heart failure [55, 56]; and elevated cardiovascular risk, morbidity and mortality [57]. These observations suggest that a complex interplay exists between the MetS and TD which results in these two pathologies frequently presenting together [58].

Although the incidence of testosterone deficiency has previously been associated with increasing age [28], stepwise regression analyses have since revealed that the effects of age on circulating testosterone are likely mediated by an increase in age-related co-morbidities, rather than age *per se* [32].

The frequent clinical presentation of the metabolic syndrome and testosterone deficiency in males warrants the development of appropriate models which represent the cardiometabolic profiles of patients whom we propose should be diagnosed as having “Testosterone-deficient MetS (TDMetS)”. Furthermore, the present study provides evidence that corroborates clinical data linking both testosterone deficiency and metabolic syndrome to adverse cardiovascular risk and outcomes.

### 4.2 Associations between obesity, the metabolic syndrome and testosterone deficiency

The reciprocal interplay between obesity and sex hormone dysfunction has been thoroughly described elsewhere [27] i.e. testosterone plays a role in the regulation of adiposity and adiposity plays a role in the regulation of sex hormone production. Briefly, the aromatase enzyme is highly expressed in adipose tissue. Consequently, in the obese phenotype, testosterone is more likely to undergo aromatisation leading to further inhibition of testosterone biosynthesis via the central hypothalamus-pituitary-gonadal feedback mechanism [59]. Inversely, obesity-induced elevations in insulin [18] and leptin [60] are features of the metabolic syndrome and individually exert suppressive effects on steroidogenesis in the testes [27]. A recent study demonstrated that diet-induced obesity in male rats suppressed the reproductive hormone axis after 9+ months of feeding [25], albeit without characterising the cardiometabolic profile of the model.

#### 4.2.1 Role of metabolic syndrome in the aetiology of testosterone deficiency

In the clinical setting, the metabolic syndrome has been strongly associated with low circulating testosterone concentrations in males [49]. Various observational studies of post-pubertal men have revealed inverse associations between circulating testosterone levels and individual components of the metabolic syndrome: 1) measurements of adiposity [50, 61]; 2) circulating triglycerides [52, 62, 63]; 3) total cholesterol/LDL [51, 64]; and 4) fasting plasma glucose/type 2 diabetes [53, 65].

In the present study, diet-induced MetS resulted in testicular atrophy in sham-operated OGD rats. Although this was not associated with reductions in circulating testosterone, this data provides evidence that MetS may result in direct atrophic effects in the testes, likely mediated by increased adipokine signalling [60]. The orchiectomy performed in OGD+ORX rats resulted in an appropriately testosterone-deficient animal model, despite these changes not being induced by the MetS *per se*.

#### 4.2.2 Role of testosterone deficiency/deprivation in the aetiology of the metabolic syndrome

Androgens increase lean body mass and decrease fat mass [66], whereas androgen deprivation therapy and androgen blockade increase fat mass and decrease lean mass [67, 68]. The role of testosterone in supressing adipogenesis [69] and improving body composition has been demonstrated by using testosterone replacement therapy in both TD [24] and obese individuals [70]. Together these observations support a role for TD in the development of weight-gain and obesity, particularly in middle-aged to older populations.

The treatment of patients with prostate hyperplasia with androgen deprivation therapy increases circulating triglycerides [71, 72] and cholesterol [73]. Similar associations were reported with anti-androgen therapy coinciding with the onset of pre-diabetic insulin resistance [68, 72, 74–76] and diabetes [76, 77], characteristics of the MetS.

In the present study, serum triglycerides, cholesterol, insulin and glucose did not change with orchiectomy-induced TD in the ORX rats. However, overt hypertriglyceridemia, hypercholesterolemia and glucose intolerance, hallmarks of the MetS, were outcomes observed in orchiectomised rats challenged with the obesogenic diet. Since glucose intolerance was not seen in either the OGD or ORX models, we infer that both the TD and MetS induced in our OGD+ORX model each partly contributed to this diabetic phenotype; and their combination resulted in a robust representation of the typical clinical presentation of the MetS.

### 4.3 Model comparisons to the clinical presentation of testosterone-deficient metabolic syndrome (TDMetS)

Rats reach sexual maturity at ~6 weeks of age [78]. In the present study, rats were 12 weeks of age when feeding commenced with either the standard rat chow or OGD for the remainder of the study (28 weeks). Rats had been sexually mature for ~14 weeks before undergoing either sham or orchiectomy (ORX) procedures at 20 weeks of age, as to avoid developmental complications in these animals.

#### 4.3.1 Obesity, dyslipidaemia and insulin resistance/diabetes and the cardiovascular system

In the current study, feeding of the obesogenic diet was associated with increased body mass; total, subcutaneous and visceral fat mass; serum triglycerides and cholesterol; and indices of glucose intolerance as well as testicular atrophy in both sham- and ORX-operated rats. The OGD-induced elevations in total fat mass resulted in fat tissue constituting more than half (57%) of the total body mass of OGD+ORX rats. Across multiple populations [79, 80], male patients presenting with a total fat mass >25% are classified as obese, with incremental increases in fat mass percentage associated with increasing risk of developing type 2 diabetes (T2DM) [81]. Additionally, visceral fat mass is independently associated with decreased insulin sensitivity [82], likely mediated by the hyperlipidemic consequences of energy utilisation from this fat depot [26].

In the present study, the hyperglycemia reported in OGD+ORX animals was partly attributed to the increased visceral adiposity and serum triglycerides in these animals. Additionally, OGD+ORX rats suffered sarcopenia as demonstrated by a loss of almost one-fifth of lean mass. This lean mass loss is also an independent risk factor for insulin resistance and dysglycemia [83].

Additionally, OGD+ORX animals were revealed to be insulin-resistant (confirmed by HOMA-IR). This insulin insensitivity and associated hyperglycemia indicated that these animals had developed insulin-resistant diabetes. It is also likely that the OGD animals although not observed to be insulin-resistant, were similarly diabetic and had likely begun to experience pancreatic β-cell dysfunction.

The OGD+ORX animals of our study were therefore viscerally obese and glucose intolerant/diabetic; and strongly representative of patients with an increased risk of cardiovascular disease and acute myocardial infarction incidence [84]. Additionally, the glucose intolerance observed in both the OGD and OGD+ORX groups likely contributed to the impaired myocardial tolerance to ischemia-reperfusion in these animals [26].

#### 4.3.2 Testosterone deficiency and myocardial ischemia-reperfusion injury

In the present study, circulating CK-MB levels were elevated with orchiectomy in both the lean and obese animals demonstrating a decreased myocardial tolerance to I-R. Recent animal studies have highlighted the role of testosterone in myocardial tolerance to ischemia (assessed by myocardial infarct size), where orchiectomised male rats also sustain greater myocardial damage following I-R insult [42]. In conjunction with these studies, our data further validate the development of models with TD in order to investigate the efficacy of cardiotherapeutics in individuals with TD-related intolerance to I-R.

Whilst cardiovascular disease mortality rates vary significantly worldwide, evidence over previous decades has consistently demonstrated that post-myocardial infarction mortality rates are twice as high in males when compared to females [85]. Although testosterone was traditionally implicated in the aetiology of atherosclerosis development, it is now proposed that the testosterone-deficient state in older males may be responsible for worsened outcomes following myocardial ischemia [86]. In fact, recent evidence suggests that after adjusting for age and comorbidities, women are more likely than men to experience myocardial infarction, heart failure, stroke and cardiovascular death [87].

There is evidence to suggest that a lack of testosterone may contribute to an adverse ischemic threshold [88, 89] which may, in part, account for the increased serious cardiovascular morbidity in patients treated with androgen deprivation therapy [57]. Conversely, more recent work has identified an increased risk of myocardial infarction within 90 days of initiating testosterone replacement therapy (TRT) [90]. However, particular limitations of this recent study were: 1) serum testosterone levels were not reported; and 2) controls included patients receiving vasodilatory phosphodiesterase inhibitors for erectile dysfunction, which may have had confounding therapeutic benefits [91].

Testosterone’s effect on myocardial tolerance to ischemia-reperfusion is better described when considering the action of enzymes that metabolise testosterone and the actions of testosterone’s metabolites. The surgical induction of hypogonadism and the subsequent reduction in circulating sex hormones impairs cardiac tolerance to I-R and increases expression of both aromatase and 5α-reductase in the rat myocardium [42] and is likely to have similar effects in other tissues (e.g. adipose). Bell and colleagues [92] showed that hearts from aromatase knockout animals demonstrate improved tolerance to I-R, suggesting that androgens may elicit cardioprotective effects independently of estrogens. These observations however conflict with others’ [42] where chemical aromatase inhibition with 4-hidroxyandrostenedione ameliorated the cardioprotection elicited by testosterone treatment in orchiectomised rats. The latter group also showed that DHT impairs myocardial tolerance to ischemia, demonstrated by both DHT treatment and by an improvement in testosterone-induced cardioprotection with finasteride (5α-reductase inhibitor) co-treatment in orchiectomised male rats.

Our data support the notion that testosterone deficiency results in adverse cardiac physiology. Both with and without the cardiometabolic dysregulation conferred by the feeding of the obesogenic diet, TD resulted in impaired myocardial tolerance to ischemia-reperfusion.

Importantly, the roles of individual sex hormones in cardioprotection during I-R remain a contentious issue amongst research groups. However, it is likely that the use of testosterone-deficient animal models with increased adiposity and metabolic dysregulation, such as the OGD+ORX model described here, will provide much needed insight into the cardiovascular implications of this clinically relevant phenotype. Subsequently, this comprehensively described model may have broader applications for the investigation of new cardiotherapeutic interventions which target one or more components of TDMetS.

### 4.4 Study limitations

#### 4.4.1 Animal age

In the present study, the animals assessed were the human equivalent of approximately 25–30 years of age and were not age-matched to the human populations being modelled (approximately 40+ years).

#### 4.4.2 Conservative sample sizes

The intentionally conservative sample sizes used in the present study potentially may have allowed for the occurrence of type II error in our statistical analyses. However, this study demonstrates that relatively small experimental groups (n = 6) can be used to comprehensively represent human pathologies (i.e. metabolic syndrome, testosterone deficiency and testosterone-deficient metabolic syndrome) with significantly different biometric profiles and characteristics. The cardiac structure and function reported in the current study described smaller experimental groups (n = 4) and, although relatively precise grouping was achieved for assessments of EF% and FS% (the primary functional outcomes assessed) the variation coefficients observed for SV and CO were as large as 12%, potentially warranting future investigation with larger animal groups.

#### 4.4.3 Myocardial injury findings

Although our findings confirm that both OGD-feeding and ORX independently impair myocardial tolerance to ischemia-reperfusion, no significant difference was observed between OGD and OGD+ORX; or ORX and OGD+ORX animals. This may be partly due to a theoretical upper-limit of CK-MB released by the moderate regional ischemia induced in these ventricles. Further investigation is required in order to confirm that effective treatment of the TDMetS would necessitate a multi-faceted approach to therapeutic intervention. Additionally, the quantification of cardiac infarct sizes may be required to confirm the significance of the CK-MB data presented in this study.

### 4.5 Study implications for animal use in research

The techniques used to establish the models in the present study are thoroughly described in an effort to facilitate the further refinement and reduction of the use of animals in research, particularly within the field of cardiovascular disease.

### 4.6 Conclusions

The current study presents the first known experimental evidence demonstrating an adverse cardiovascular risk profile and impaired tolerance to ischemia-reperfusion in testosterone-deficient animals with diet-induced metabolic syndrome. The model of combined pathology used in this study is strongly representative of patients presenting with a combination of testosterone deficiency and the metabolic syndrome. In conjunction with the abundance of clinical data supporting the existence of a condition that could be described as “testosterone-deficient metabolic syndrome” or “TDMetS” this study provides additional evidence that the combination of these two conditions may exacerbate cardiometabolic risk factors. Accordingly, we propose that future animal-based investigations of cardiovascular disease should utilise appropriate models of the metabolic syndrome and hypogonadism.

## Supporting Information

S1 Checklist Animal Research: Reporting of *In Vivo* Experiments (ARRIVE) Guidelines Checklist. (PDF)
